# Influence of Cholesterol on the Regulation of Osteoblast Function

**DOI:** 10.3390/metabo13040578

**Published:** 2023-04-21

**Authors:** Alena Akhmetshina, Dagmar Kratky, Elizabeth Rendina-Ruedy

**Affiliations:** 1 Gottfried Schatz Research Center, Molecular Biology and Biochemistry, Medical University of Graz, 8010 Graz, Austria; 2 Department of Medicine, Division of Clinical Pharmacology, Vanderbilt University Medical Center, Nashville, TN 37232, USA; 3 BioTechMed-Graz, 8010 Graz, Austria; 4 Molecular Physiology and Biophysics, Vanderbilt University, Nashville, TN 37235, USA

**Keywords:** osteoblast, cholesterol, bone metabolism, osteoporosis, statins, cholesterol metabolites, osteoblastogenesis

## Abstract

Bone is a dynamic tissue composed of cells, an extracellular matrix, and mineralized portion. Osteoblasts are responsible for proper bone formation and remodeling, and function. These processes are endergonic and require cellular energy in the form of adenosine triphosphate (ATP), which is derived from various sources such as glucose, fatty acids, and amino acids. However, other lipids such as cholesterol have also been found to play a critical role in bone homeostasis and can also contribute to the overall bioenergetic capacity of osteoblasts. In addition, several epidemiological studies have found a link between elevated cholesterol, cardiovascular disease, an enhanced risk of osteoporosis, and increased bone metastasis in cancer patients. This review focuses on how cholesterol, its derivatives, and cholesterol-lowering medications (statins) regulate osteoblast function and bone formation. It also highlights the molecular mechanisms underlying the cholesterol–osteoblast crosstalk.

## 1. Introduction

The bones that constitute the skeleton (approximately 15% of the body weight) are one of the largest organs in humans and animals. Bone tissue is a type of connective tissue characterized by strength and structural support. As external forces are constantly changing, bone maintains its supportive properties by continuous turnover, although this rate is influenced by both genetic and environmental factors. In this regard, bone is inherently dynamic in nature, as it is being degraded and regenerated [1,2]. This process occurs through primary bone cells represented by bone-forming osteoblasts, bone-resorbing osteoclasts, and terminally differentiated mechano-sensing osteocytes [3], which are deeply embedded in the extracellular matrix (ECM) and the mineralized portion of the bone [4]. Thus, osteoblasts form bone by orchestrating the secretion of matrix and mineralization vesicles that give rise to ‘bone’. This unique composition of bone is dominated by the inorganic components that constitute the mineralized portion, including hydroxyapatite [Ca_10_(PO_4_)_6_(OH)_2_], owing to the hard and brittle characteristics of bone, while the organic portion contributes to its plasticity [5,6]. Actual bone composition is more complex, and further studies are required to find specific nanocomposites that may play a role as scaffolds in bone regeneration [7].

As the main players responsible for bone formation, osteoblasts differentiate from multipotent mesenchymal stem cells of the bone marrow [8] with the support of the transcription factors RUNX2 and Osterix (encoded by *Sp7*) [9]. The differentiation of pre-osteoblasts into osteoblasts is accompanied by the formation of new capillaries [10]. At high partial pressure of oxygen (pO_2_) values, osteogenic cells differentiate into osteoblasts, whereas at a low pO_2_ they differentiate into chondroblasts [11]. During active bone formation, three types of osteoblasts are distinguished, whose ultrastructure reflects the peculiarities of their functional activity [3,12]. Osteoblasts are further divided into mature and immature [13], active and quiescent cells [14]. Mature osteoblasts are characterized by high osteogenic activity, rapidly producing organic ECM, including collagen type 1, proteoglycans, and osteocalcin (OCN) [3,15]. Immature osteoblasts adhere directly to the bone surface within the periosteum and endocortical surface, and their cytoplasm contains low concentrations of glycogen granules [3]. The primary function of active osteoblasts is the synthesis of organic bone matrix components, cytokines, and growth factors, as well as the production of matrix vesicles, which participate in bone tissue mineralization. To this end, the primary markers of osteoblasts are proteins involved in matrix production and mineralization, including the ectopic enzyme alkaline phosphatase (ALP), as well as osteonectin, and OCN [3,15].

Bone formation requires many endergonic, or energy-depleting, reactions. Examples of these energy-dependent processes include chromatin remodeling [16,17], actin organization [18,19], ECM and mineralization vesicle secretion [20,21], active transport [22,23,24], lysosomal acidification [25], and ALP activity [26]. Therefore, adequate energy metabolism is essential for overall osteoblast function and bone quality [27]. Cellular energy is derived from the hydrolysis of adenosine triphosphate (ATP) generated either by glycolysis in the cytoplasm or by oxidative phosphorylation in mitochondria [22,28]. Several studies have described a preference for oxidative phosphorylation to counteract the deleterious effects of reactive oxygen species (ROS) in mesenchymal stem cells that have differentiated into an osteoblast lineage [29,30]. However, in mature osteoblast cells, such as the MC3T3-E1 cell line and primary bone marrow stromal cells (BMSCs), glycolysis was preferred over oxidative phosphorylation for energy production [23,31,32,33,34]. It remains unclear why these cells switch from oxidative phosphorylation, an ATP-proficient pathway, to aerobic glycolysis, which is inefficient in ATP generation. Due to the presumed hypoxic environment within the skeletal niche, the production of the pentose phosphate pathway intermediates and reduced ROS production have also been suggested as possibilities [29,35]. Finally, it is important to emphasize that the ATP required for bone formation is finite and limited. Altering metabolic processes outside of oxidative phosphorylation and glycolysis can have a profound impact on cellular function by shifting ATP requirements and/or intermediates, thereby altering overall bioenergetics. Therefore, we propose cholesterol homeostasis as an additional regulator of osteoblast bioenergetic capacity.

As mentioned previously, ATP generation requires the catabolism of substrates, of which osteoblasts have been shown to utilize glucose, glutamine, and fatty acids for energy production. While the crucial role of these substrates has been demonstrated for decades and confirmed by recent sophisticated studies [28,30,31,34,36,37], other metabolic pathways remain less understood. Of particular interest are the effects of lipids and lipid metabolism on osteoblast function. For example, cholesterol, along with endogenous metabolites (e.g., prostaglandins, oxysterols) and several specific lipids derived from membrane phospholipids (i.e., sphingosine-1-phosphate, lysophosphatidic acid, and various fatty acid amides), are gaining increasing scientific interest concerning bone and its regulation of cellular energy status. For the purposes of the current review, cholesterol, a single-atom cyclic hydrophobic alcohol, is critical for many cellular processes [38], both directly and indirectly. For example, in terms of osteoblast function, cholesterol has been reported to be essential for survival and function, bone mineralization, and critical signaling pathways [39]. Additionally, the manipulation of cholesterol status could alter osteoblast bioenergetics and shift various metabolic substrates.

**Figure 1:**
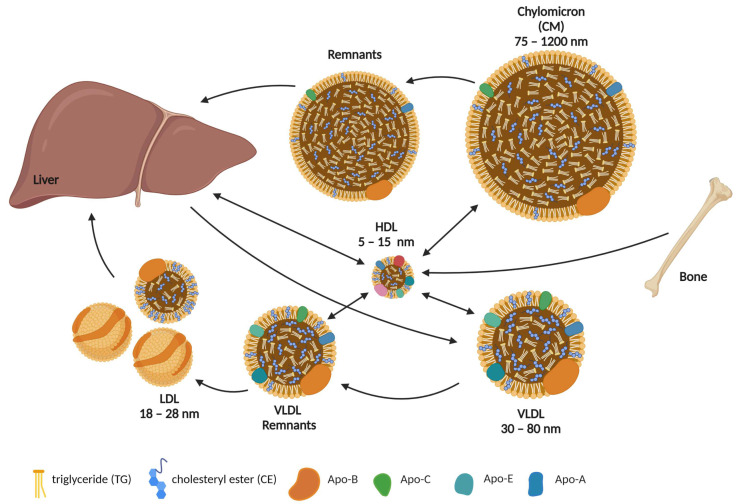
Systemic cholesterol flux. Chylomicrons (CM) are formed in the lymphatic system of the intestinal villi. They package and transport up to half of all triglycerides (TG) and lymph cholesterol. As peripheral lipid uptake progresses, CM lose most of their TG to various cells, whereas the relative content of cholesterol and its esters (CE) increases and their size decreases. CM remnants are taken up by the liver, where CE and TG are eventually hydrolyzed. Very low-density lipoproteins (VLDL) carry TG, phospholipids, cholesterol, and CE from the liver to other tissues. As TG are broken down, the diameter of VLDL decreases and they become low-density lipoproteins (LDL). High-density lipoproteins (HDL) ensure the reverse transport of cholesterol from extrahepatic tissues, such as bone, back to the liver. Important structural components of lipoproteins are the apolipoproteins (Apo), the types of which vary but remain specific to certain types of lipoproteins. This figure was created using Biorender.com (accessed on 24 March 2023).

Cholesterol is a non-polar hydrophobic molecule that is transported in circulation as part of lipoprotein particles. Maintaining systemic cholesterol homeostasis is determined by endogenous biosynthesis, uptake, efflux, transport, storage, utilization, and/or excretion [40]. Low-density lipoproteins (LDL) transport the majority of cholesterol in the plasma from the liver to peripheral tissues and cells, including osteoblasts. Reverse cholesterol transport describes cholesterol transport in high-density lipoproteins (HDL) from peripheral tissues back to the liver for secretion into the bile [41] (Figure 1).

**Figure 2:**
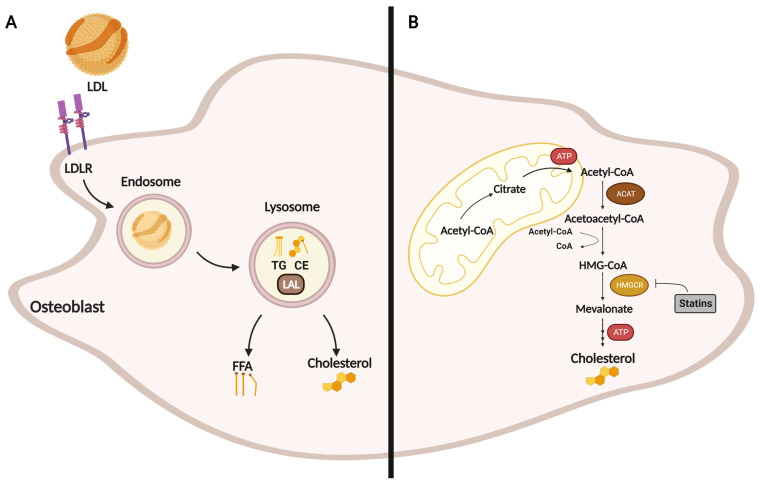
Osteoblast cellular cholesterol homeostasis. (**A**) Exogenous LDL uptake in osteoblast via LDL-LDLR endocytosis is an important source of CE and TG for lysosomal hydrolysis. Lysosomal acid lipase (LAL)-mediated lipid catabolism leads to the release of free cholesterol and free fatty acids (FFA) for further cellular needs. (**B**) Cholesterol formation begins with acetyl-coenzyme A (CoA), two molecules of which are converted into acetoacetyl-CoA by acetyl-CoA acetyltransferase (ACAT). The first step in the mevalonate pathway from 3-hydroxy-3-methylglutaryl (HMG)-CoA to mevalonate is catalyzed by HMG-CoA reductase (HMGCR), which is the main target of statins. This figure was created using Biorender.com (accessed on 13 March 2023).

Like many other cell types, osteoblasts derive free cholesterol by exogenous uptake via LDL-LDL receptor (LDLR) endocytosis and/or by de novo biosynthesis. In this regard, circulating LDL particles interact with the osteoblast surface and are internalized by LDLR-mediated endocytosis. These endosomes fuse with the lysosome inside the cell, where lysosomal acid lipase (LAL) hydrolyzes CE and TG to form free cholesterol (FC) and free fatty acids (FFA) (Figure 2A). Apart from the LDL-LDLR uptake of cholesterol, osteoblasts can also use the endogenous cholesterol biosynthetic pathway (Figure 2B). To do so, these cells must utilize acetyl-CoA precursors to initiate the mevalonate pathway. The formation of acetoacetyl-CoA from two molecules of acetyl-CoA generated by the β-oxidation pathway is the first step in de novo cholesterol biosynthesis, followed by the formation of β-hydroxy-β-methylglutaryl-CoA (HMG-CoA) from acetoacetyl-CoA and a third acetyl-CoA molecule by HMG-CoA synthase. The enzyme HMG-CoA reductase (HMGCR) then reduces this molecule to mevalonate. Mevalonate production is the rate-limiting and irreversible step in cholesterol biosynthesis that can be inhibited by statins (a class of cholesterol-lowering drugs).

Osteoblasts contain machinery to regulate cholesterol via both LDL-LDLR endocytosis as well as via the cholesterol biosynthetic pathway. While cholesterol is needed for many cellular processes, cellular energy or bioenergetics can be altered based on which pathway is activated. Therefore, the current review aims to highlight recent findings on the relationship between cholesterol metabolism and the formation, differentiation, and function of osteoblasts as the most represented cell population in bone, both under normal physiological conditions and in pathologies.

## 2. Role of Cholesterol in Osteoblast Formation, Function, and Metabolism

Publications demonstrating the importance of cholesterol in the bone formation process and metabolism date back to the late 1990s and early 2000s. At that time, authors successfully showed that the osteoblastic differentiation of mouse bone-marrow-derived pluripotent M2–10B4 stromal cells is regulated by the inhibition of cellular cholesterol biosynthesis through the rate-limiting enzyme HMGCR. They also confirmed that cholesterol and its metabolic intermediates are required for the maturation of bone-marrow-derived mesenchymal stem cells (MSCs) [42]. In line with these studies, the induction of osteoblastic differentiation and mineralization was confirmed in M2–10B4 treated with cholesterol-derived oxysterols [43]. Further studies revealed a beneficial effect of cholesterol treatment on mouse mesenchymal stem cells resulting in increased cell number and the formation of mineralized nodules, identified by an increase in ALP together with other important osteoblast differentiation genes such as bone morphogenetic protein-1 (*Bmp-1*), *Runx2*, and *Bglap2* (*gene encoded* OCN). Additionally, this study indicated an essential role of CE in osteogenesis by inhibiting acyl-CoA: cholesterol acyltransferase (ACAT) [44].

While cholesterol clearly affects osteoblast function, the effect of cholesterol on bones in general and on osteoblasts themselves appears to be dose- and/or time-dependent. For instance, MC3T3-E1 osteoblasts showed reduced proliferation and differentiation rates as well as increased oxidative damage when treated with high concentrations of cholesterol [45]. In addition, the treatment of osteogenic cells in bone marrow stroma with minimally oxidized low-density lipoprotein (MM-LDL) as a source of cholesterol resulted in reduced differentiation. By impairing ALP activity, collagen processing, and mineralization, MM-LDL might interfere with key stages of differentiation, most probably by triggering peroxisome proliferator-activated receptor α and mitogen-activated protein kinase-dependent signaling pathways [46].

One of the major HDL protein constituents, apolipoprotein A-I (apo A-I), is a crucial modulator of plasma cholesterol trafficking and cellular cholesterol balance [47]. Interestingly, mesenchymal stem cells from apo A-1-deficient mice differentiate less into osteoblasts and more into adipocytes, indicating a switch of their subtypes toward adipocyte progenitors [48]. Osteoprogenitor cells, calvarial cells, embryonic fibroblasts, and primary BMSCs treated with an osteogenic oxysterol combination blocked and reversed the inhibition of osteogenic differentiation caused by xanthine/xanthine oxidase (XXO) and MM-LDL. The ability of oxysterols to positively regulate osteogenic differentiation and overall osteogenic properties was associated with cyclooxygenase 1 and MAPK-dependent mechanisms against oxidative stress [49]. Oxidized LDL (oxLDL), which has been shown to play a critical role in the initiation and progression of atherosclerosis, leads to elevated levels of the receptor activator of NFkappaB ligand (RANKL) in UMR106 rat osteoblasts and human osteoblast-like cells (MG63 human osteosarcoma cell line), which has been linked to osteoclastogenesis and bone resorption [50,51]. Moreover, oxLDL inhibits the differentiation and mineralization of osteoblasts via phosphate signaling and phosphate-induced mineralization [51], demonstrating the deleterious impact of these particles on bone development. Recent data additionally hint that osteoblast demineralization driven by oxidized HDL (oxHDL) occurs because of inflammation [52].

These in vitro data were confirmed by experiments in rodents fed a high-cholesterol diet. Both C57BL/6 and C.B-17/Icr-SCID/Sed-Prkdcscid (severe combined immunodeficiency) mice fed a high-fat high-cholesterol diet (40 kcal% fat, 1.25% cholesterol) exhibited an osteoporotic bone phenotype, along with elevated osteoclast number, trabeculae loss, and thinning of trabeculae and cortex [53]. Female mice fed a Western diet (1.1 mg cholesterol/g diet) exhibit a drastic phenotype with low bone mass [54]. In C57BL/6 mice fed an atherogenic diet (high-fat/high-cholesterol plus sodium cholate), a reduction in bone mineral content and mineral density (BMD) of the femur, estimated by peripheral quantitative computed tomographic scanning and confirmed by lower OCN expression, was observed compared with mice fed a meat-supplemented diet [55]. As expected, a high-cholesterol diet caused hypercholesterolemia and, interestingly, was associated with increased bone resorption and decreased bone formation, leading to reduced BMD in rats [45].

The role of lipoproteins was identified in several physiological and pathological conditions of bones. Ldlr−/− mice showing severe hypercholesterolemia [56] became an important model for the study of bone metabolism. Reduced ALP activity and a delayed Ca^2+^ incorporation suggest defective osteoblastogenesis in cells isolated and differentiated from Ldlr−/− mice compared to wild-type littermates. Moreover, the expression of *Runx2* and *Osterix*, key factors in osteoblastic differentiation, was decreased [57]. Scavenger receptor class B, type I (SR-BI)*,* a receptor involved in the uptake of CE from HDL, encoded by *Scarb1* gene, was found to play a role in bone formation. Unexpectedly, Scarb1−/− mice, which tend to develop atherosclerosis, exhibit higher bone mass. The authors proposed that SR-BI may influence proliferation and differentiation processes in MSCs and thus contribute to bone turnover by modulating adrenocorticotropin (ACTH)/glucocorticoids levels through cholesterol uptake in the adrenal glands [58]. However, the high bone mass in these mice might be caused by a rise in serum ACTH, which has anabolic effects on osteoblasts [59,60,61].

Recently, our group has shown that LAL, as the only known essential CE hydrolysis enzyme, is essential for proper osteoblast metabolism [62]. Loss of LAL is associated with increased CE and TG accumulation in multiple cells and tissues [63,64]. Global Lal−/− mice exhibited lower cortical bone thickness and strength as well as fewer osteoblasts, whereas osteoclast numbers were unchanged. Impaired osteoblastogenesis in these mice was connected to Wnt, Notch, and BMP signaling pathways and resulted in drastically altered lipid metabolism [62].

Further data hint at a delay in skeletal development in utero in pregnant C57BL/6J and Swiss Albino mice exposed to a high-cholesterol diet. The animals suffered from a decrease in total length and mineralized bone due to the inhibition of bone formation and increased resorption during bone remodeling [65]. Additionally, these mice suffered from permanent bone growth deficiencies not only during pregnancy but also during lactation, with no recovery later in life. In offspring of both C57BL/6J and Swiss Albino mice, dramatic changes in bone microarchitecture and osteogenic gene expression profile were observed, even when the mice were switched to a regular diet [65]. Furthermore, the cholesterol metabolite 27-hydroxycholesterol (27-HC) at high concentrations in mouse serum caused a significant BMD decrease [66]. Studies disclosed that 27-HC reduces bone deposition while increasing bone turnover by competing with the 17β-estradiol receptor and activating liver X receptors [67]. Research studies carried out in cells and animal models to evaluate the influence of cholesterol and its different sources and derivates on osteoblast cell proliferation, differentiation, and functions are summarized in Table 1.

Human studies sparked interest in the role of cholesterol in bone metabolism, as it has been repeatedly observed that a high-cholesterol diet affects bone health, particularly with regard to high cholesterol levels and osteoporosis. Specific attention was paid to postmenopausal bone turnover and its correlation with increased serum LDL-cholesterol levels, which were linked to bone loss and increased bone fracture risk [68,69,70,71,72,73].

Like mice, patients with hypercholesterolemia showed increased bone turnover associated with elevated serum bone-specific alkaline phosphatase and N-terminal telopeptide of type I collagen, which in turn correlated positively with total and LDL cholesterol [74,75]. Subjects with the familial apolipoprotein B-10 R3500Q variation were found to have low BMD in the femoral neck and lumbar spine [76]. Unexpectedly, patients affected by heterozygous familial hypercholesterolemia (FH) caused by *LDLR* gene mutations had comparable BMD at the femoral neck and markers of bone resorption. However, the authors noted that serum OCN, urinary calcium, and glomerular filtration rate were negatively correlated with a higher degree of aortic calcification [77]. Even more controversial data were found in the Spanish male population, exhibiting increased BMD in femoral neck, total hip, and lumbar spine [78]. Clinical studies have shown that high LDL, HDL, and total cholesterol levels were associated with low total lumbar, femur neck, and total hip BMD in men and women with type 2 diabetes. Likewise, cholesterol concentrations were negatively related with OCN, procollagen type I N-terminal propeptide, and β-crosslaps [79]. While some of the clinical data remain controversial, elevated cholesterol generally appears to exert a negative impact on skeletal health.

Taken together, these data suggest that cholesterol is an important player in osteoblastogenesis and adequate bone formation, as well as bone metabolism. However, high cholesterol concentrations are negatively associated with skeletal health, as evidenced by increased resorption and decreased bone formation. However, whether hyperlipidemia is a consistent cause of low BMD and high fracture risk in humans has not been established.

## 3. Effect of Statins on Osteoblasts

Although the liver and intestine produce more than 80% of the body’s total daily cholesterol, bone cells also participate in cholesterol synthesis via the mevalonate pathway [80]. As mentioned above, HMGCR catalyzes the key step in the synthesis of mevalonic acid, which is a precursor of sterols, isoprenoids, and other lipids [81]. Statins are a commonly prescribed medication to lower cholesterol that act via the inhibition of HMGCR. Interestingly, these drugs also possess bone-protective properties. As such, simvastatin and lovastatin have been demonstrated to promote bone growth associated with the upregulation of bone morphogenetic protein -2 (BMP-2) in mice and rats [82]. Further studies revealed that osteoblast cells and MSCs treated with simvastatin and lovastatin increased ALP activity, the expression of collagen and osteopontin, and upregulated BMP-2 expression and nodule formation [44,45,83,84,85,86]. In fact, lovastatin activates AKT in osteoblasts by promoting tyrosine phosphorylation at the p85 regulatory subunit of phosphatidylinositol 3-kinase [84]. A study on the mechanism of action of statins revealed that statins enhance osteoblast-activating osteoprotegerin and inhibit the mRNA expression of CD9, M-CSF, and RANKL by activating p38MAPK and blocking the Ras/ERK pathway, thereby having a major influence on osteoclast activity [87]. Additionally, Rho and MKP-1 are rendered inactive by the reduction in farnesyl pyrophosphate synthesis via reducing GR production, which may reverse their detrimental effects on osteogenesis. Statin-activated TGF/SMAD3 suppresses osteoblast apoptosis, while the estrogen receptor inhibits osteoclastogenesis by activating the osteoprotegerin/RANKL/RANK system [88]. Thus, the stimulation of osteoblast differentiation, suppression of osteoblast death, and inhibition of osteoclastogenesis are some of the effects of statins on bone anabolism.

## 4. Mechanism of Osteoblast Regulation by Cholesterol via Signaling Pathways

### 4.1. Wnt-Lrp5-β-Catenin

One of the main components of the lipid oxidation system in osteoblasts is Wnt-Lrp5 signaling [27]. *Lrp5*, encoding low-density lipoprotein receptor-related protein 5, causes the autosomal recessive disorder osteoporosis-pseudoglioma syndrome (OPPG), which is characterized by low bone mass, increased fractures, and deformation in patients [89]. It is appreciated that the name of Lrp5 inherently implies its involvement in ‘lipoproteins’. This includes its primary ligand, Wnt, which is heavily lipidated. LRP5 itself is involved in fuel metabolism, which was confirmed in several studies linking polymorphisms of this gene to hypercholesterolemia [90,91,92,93] and the reduced clearance of CR from the circulation [94]. In addition, the high-bone-mass trait (HBM) mutation, which confers unique osteogenic activity in bone remodeling, is also associated with the chromosomal region corresponding to *LRP5* [95]. Mice lacking Lrp5 specifically in terms of osteoblasts and osteocytes exhibit a decrease in postnatal [36] and adult bone mass and also show an increase in body fat with a corresponding reduction in whole-body energy expenditure [27,36].

The regulation of osteoblast metabolism by cholesterol was also connected to Wnt/β-catenin, as recent findings suggested that cholesterol is crucial in the modulation of Wnt/β-catenin for adult skeleton development and the regulation of adult synovium, articular cartilage, and osteoblasts [96]. Furthermore, the knockdown of forkhead box protein f1 gene (*Foxf1*), an activator of the Wnt/β-catenin signaling pathway, induced osteogenesis and significantly boosted the expression of genes associated with osteoblasts, ALP activity, and mineralization in vitro [97]. This effect was partially restored by the Wnt signaling inhibitor Dickkopf-related protein 1. Moreover, *FOXF1* upregulation in bone tissue of postmenopausal osteoporosis patients was shown to be associated with decreased bone mass and bone growth [97].

### 4.2. TGF-β/BMP2

**Figure 3:**
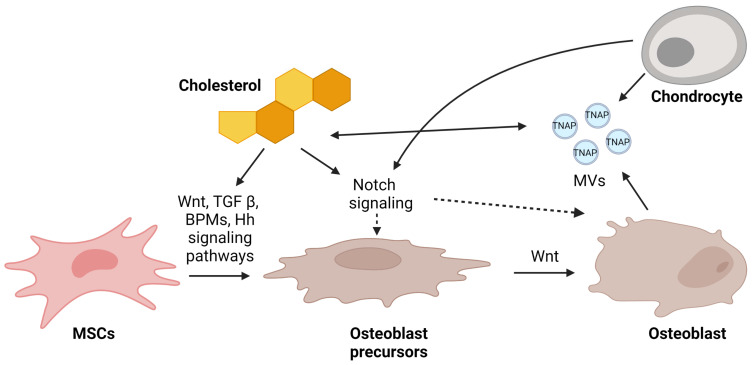
Possible mechanisms of regulation of osteoblastogenesis by cholesterol. In response to Wnt ligand, BMPs, and TGFβ, bone-marrow-derived mesenchymal stem cells (MSCs) differentiate into mature osteoblasts. All these pathways were found to be regulated by cholesterol. On the other hand, sufficient release of matrix vesicles (MVs) by chondrocytes and osteoblasts ensures appropriate mineralization. Chondrocytes and cholesterol are connected via the Notch signaling pathway, and it is still unclear whether this directly affects pre-osteoblasts or osteoblasts. Nevertheless, this pathway is essential for proper bone formation. This figure was created using Biorender.com (accessed on 13 March 2023).

The gene expression profile of rats fed a high-cholesterol diet revealed some downregulated genes, including *Wnt5*, β-catenin, *Tgfbr*, *Smad4*, *Smad6*, *Smad7*, *Bmpr2*, and *Bmp6*, which are known to be involved in the TGF-β/BMP2 and Wnt signaling pathways (Figure 3). The downregulation of these genes promoted the suppression of the differentiation of MSCs into osteoblasts, the maturation of osteoblasts themselves and, thus, decreased bone formation [45].

### 4.3. Notch

Historically, the Notch signaling system is essential for cellular homeostasis, cell-to-cell interaction, and cell differentiation in many tissues [98,99,100]. Therefore, Notch serves as a key biomarker for osteoblasts by participating in many signaling pathways that are important for osteoblast differentiation and function [101,102]. Interestingly, cholesterol synthesis and regulation have been suggested to play a critical role in subchondral bone formation. Osteoblast-induced culture reduced intracellular cholesterol content and cholesterol synthesis in chondrocytes [100]. These alterations were accompanied by a profound downregulation of key genes involved in chondrocytes Notch signaling [100]. Therefore, while the mechanistic link between Notch signaling and cholesterol regulation during subchondral bone formation remains unknown, osteoblasts appear to alter cholesterol synthesis and Notch signaling in chondrocytes (Figure 3).

### 4.4. Hedgehog (Hh)

Cholesterol has recently been found to be an endogenous stimulator of Hh signaling, which has been linked to the pathophysiology of osteoporosis. Using ST2 cells, a BMSC cell line, it was found that cholesterol treatment markedly decreased the expression of osteoblast marker genes (*Alpl*, *Sp7*, and *Ibsp*) along with ALP activity, stimulating the expression of *Gli1,* one of the Hh targets. However, further osteogenic differentiation of BMSCs required physiological levels of endogenous cholesterol because of Hh signaling, suggesting that both Hh-dependent and -independent processes play a role in osteoblast differentiation [103].

## 5. Mechanism of Mineralization Regulation by Matrix Vesicles

Another important mechanism of osteoblast-cholesterol crosstalk is based on matrix vesicles (MVs) (Figure 3). They are released by chondrocytes and osteoblasts for biomineralization, which involves the systematic deposition of calcium and PO_4_^3−^-containing minerals to produce a calcium phosphate salt that mimics hydroxyapatite [104,105]. Interestingly, MVs have high cholesterol, sphingomyelin, and phosphatidylserine contents, which is similar to the content of rafts in the plasma membrane [105,106]. MVs additionally contain a variety of hydrolytic enzymes, including tissue-nonspecific alkaline phosphatase (TNAP) with phosphomonohydrolytic activity [107]. Recent findings implied that TNAP catalyzed ATP hydrolysis more effectively in reconstituted liposomes when they consisted of dipalmitoylphosphatidylcholine (DPPC), dioleoylphosphatidylcholine (DOPC), DPPC:cholesterol, and DOPC:cholesterol. As a function of time, these lipid microenvironments produce more inorganic phosphate, which subsequently encourages further biomineralization. Moreover, the presence of cholesterol causes a higher exclusion pressure, showing that glycosylphosphatidylinositol anchor (GPI)-TNAP penetrates deeper into the saturated lipid monolayer, which in turn influences the action of the membrane and impacts the activity of the enzyme [106].

## 6. Conclusions

**Figure 4:**
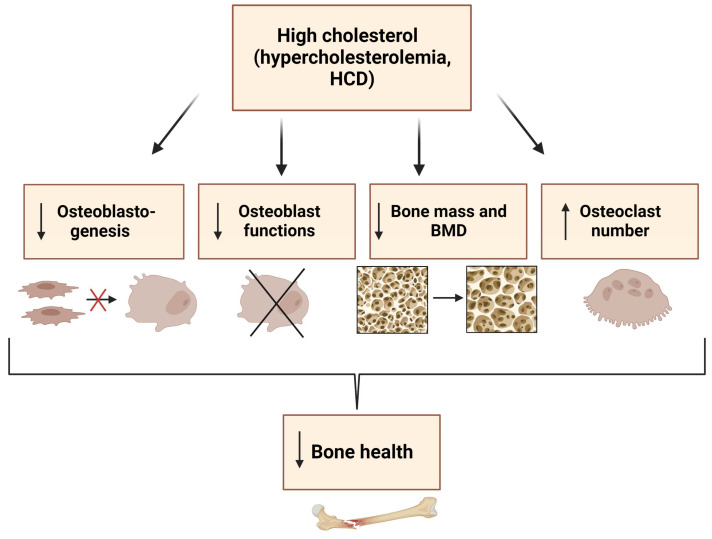
Summary of the effects of cholesterol on osteoblasts. A high cholesterol diet (HCD) and hypercholesterolemia have negative effects on bone health. They manifest as reduced proliferation and differentiation of bone marrow stromal cells, which in turn results in decreased osteoblastogenesis. Such a hyperlipidemic condition affects several aspects of osteoblast function and homeostasis, increases the number and activity of osteoclasts, and decreases bone mass and bone mineral density (BMD). This figure was created using Biorender.com (accessed on 13 March 2023).

The interaction between lipids and bone metabolism has become a hot topic, especially due to the important role both play in bone health and diseases. However, an overview of the balance between lipids and bone, especially between cholesterol and osteoblasts, has yet to be clarified. On the one hand, cholesterol and its derivates regulate osteoblast differentiation and support their cellular functions. On the other hand, high levels of cholesterol, either in the form of dietary supplements or produced endogenously, inhibit osteoblastogenesis and thus increase osteoporosis risk. The consumption of a currently quite popular high-cholesterol diet leads to an increased lack of bone growth that persists into old age and impacts overall health. The schema below summarizes the current literature on the effects of hypercholesterolemia on osteoblasts and skeletal homeostasis in rodents and humans (Figure 4). Although these data are compelling, further research should be directed toward fully unraveling the molecular mechanisms by which cholesterol alters osteoblast function.

Thus, the results of the studies covered in this review demonstrate the importance of individuals lowering their circulating cholesterol concentrations to maintain their bone health. Furthermore, the regulatory pathways highlighted in this review may become potential targets for regulating born turnover in patients at risk of or with existing hypercholesterolemia.

It is also worth mentioning that cholesterol biosynthesis is a complex process that requires 18 moles of acetyl-CoA, 36 moles of ATP (or, according to some sources, 99 molecules of ATP [108]), 16 moles of NADPH, and high oxygen consumption. This is usually higher than the cost of gluconeogenesis (4 ATP, 2 GTP, and 2 NADH) or fatty acid synthesis (8 acetyl CoA, 7 ATP + 14 NADPH) [109]. In addition, acetyl-CoA carboxylase and HMGCR activities reflect not only the cellular demand for cholesterol and fatty acids but also the availability of common substrates (particularly citrate and acetyl-CoA) [38]. The supply of and demand for these common precursors is a function of the cells’ overall metabolic activity, which may affect the energy status of the cell. This in turn may become an important topic for further consideration and research on the complex interaction between cholesterol metabolism and different cell types, especially osteoblasts.

## 7. Outlook

The general inverse relationship between cholesterol and BMD has been appreciated for some time. While some of these data remain controversial, likely due to the comorbidities associated with hypercholesterolemia, dyslipidemia often results in an increase in fracture incidence. To date, there are few molecular data delineating the mechanism(s) that regulate these processes. This review aimed to highlight how cholesterol status may impact bone formation via the modulation of osteoblast processes. Although cholesterol is not considered a classical regulator of cellular bioenergetics, cholesterol homeostasis alters ATP status. The differences in ATP demand required for cholesterol derived from LDL-LDLR endocytosis versus the biosynthetic pathway could directly influence the ability of osteoblasts to form bone. Therefore, future studies are needed to determine how ATP availability shifts from one pathway to another beyond direct substrate utilization for ATP generation. Expanding the field of osteoblast bioenergetics may open an area of research that could lead to therapeutic options to improve overall skeletal health.

## Figures and Tables

**Table 1:** Effect of cholesterol on osteoblast proliferation and functions.

Research Object	Treatment/Diet	Cholesterol/Cholesterol Derivatives Concentration	Effect	Reference
M2-10B4 (mouse stromal cell line)	22(S)-hydroxycholesterol 20(S)-hydroxycholesterol (SS)	5 mM	↑ALP activity ↑mineralization ↑OCN mRNA	[43]
MSCs (bone marrow- derived mesenchymal stem cells)	Chol:MbCD (‘‘water-soluble cholesterol’’ containing 30 mg of cholesterol/g solid)	5, 10 and 15 μg/mL	↑differentiation ↑ALP activity ↑mineralized nodules	[44]
MC3T3-E1 (mouse cell line of immature osteoblasts)	Cholesterol	0, 12.5, 25, and 50 μg/mL	↓proliferation ↓differentiation ↑oxidative injury	[45]
M2–10B4	Oxidized low-density lipoprotein (MM-LDL)	150 µg/mL	↓osteogenic differentiation ↑adipogenic differentiation	[46]
M2-10B4, Primary mouse bone marrow stromal cells	Xanthine/xanthine oxidase (XXO) minimally oxidized LDL (MM-LDL) Osteogenic oxysterol combination 22(S)- and 20(S)-hydroxycholesterol	50 mM/40 mU/mL 200 mg/mL 0.1–5 µM	↓markers of osteogenic differentiation blocked and reversed the inhibition of osteogenic differentiation	[49]
UMR106 (rat osteoblast-like cell line)	Oxidized LDL (oxLDL)	10–50 μg protein/mL	↓mineralization	[51]
MG63 (human osteosarcoma cell line)	Oxidized LDL (oxLDL)	10–50 μg/mL	↑cell-associated and extracellular RANKL levels	[50]
HOBs (primary human osteoblast cells)	Oxidized HDL (oxHDL) oxHDL with adiponectin	100 μg/mL protein 100 μg/mL; 5, 10, and 15 μg/mL	↓mineralization, ↓calcium incorporation. ↑expression of mineralization markers ↓inflammatory markers	[52]
Rat	Poly (lactic-co-glycolic acid) (PLGA) scaffolds alone or oxysterol cocktail	140 ng (low dose) 1400 ng (high dose)	slight bone healing ↑bone formation	[43]
Rat	High-cholesterol diet	77% normal diet food, 3% cholesterol and 20% lard	↓femur BMD ↓osteocalcin ↑carboxy-terminal collagen crosslinks	[45]
C57BL/6 and C.B-17/Icr-SCID/Sed-Prkdcscid male mice	High-fat/high-cholesterol (HFHC) diet	1.25% cholesterol	↓cortical and trabecular bone in the femurs and vertebrae ↓bone mineral density (BMD)	[53]
OF1 female mice	Westerntype diet	1.1 mg cholesterol/g diet	↓BMD	[54]
C57BL/6 and C3H/HeJ male mice	High-fat (atherogenic) diet	1.25% cholesterol	↓femoral and vertebral mineral content ↓BMD	[55]
C57BL6/J and Swiss Albino mice	High-cholesterol (HC) diet	0.5% cholesterol	↓osteoblast cell activity ↑osteoclast cell population delayed skeletal ossification	[65]
